# Balancing inflammation: the specific roles of serum amyloid A proteins in sterile and infectious diseases

**DOI:** 10.3389/fimmu.2025.1544085

**Published:** 2025-02-10

**Authors:** Tirthankar Mohanty, Katarina Miličević, Henri Göthert, Andreas Tillmann, Médea Padra, Praveen Papareddy, Heiko Herwald

**Affiliations:** ^1^ Infection Medicine, Department of Clinical Sciences Lund, Lund University, Lund, Sweden; ^2^ Department of Laboratory Medicine, Lund University, Lund, Sweden; ^3^ Respiratory Medicine, Allergology, & Palliative Medicine, Lund University, Skåne University Hospital, Lund, Sweden

**Keywords:** innate immunity, PAMPs (pathogen associated molecular patterns), inflammation, serum amyloid proteins, cytokines

## Abstract

Serum Amyloid A (SAA) proteins are acute-phase reactants with critical roles in sterile and bacterial inflammation. Through *in vitro* and *in vivo* experiments, we demonstrate that SAA proteins amplify cytokine and chemokine responses during sterile inflammation and enhance bacterial clearance in infectious conditions. Mechanistically, SAA proteins augment NF-κB signaling, driving pro and anti-inflammatory mediator production. *SAA^-/-^* mice carrying a deletion of the *Saa1*, *Saa2*, *Saa3*, and *Saa4* serum amyloid A genes have better survival rates in sterile sepsis but are more prone to bacterial sepsis than their *SAA^+/+^* counterparts, emphasizing their dual functionality in immune regulation. Overexpression of *Saa1*, *Saa2*, *Saa3*, and *Saa4* in macrophages enhances NF-κB-mediated pro-inflammatory cytokine production and bacterial clearance during infection. Together, our results show that SAA proteins are key modulators of inflammation, with distinct mechanisms tailored to sterile and infectious contexts.

## Introduction

The family of Serum Amyloid A (SAA) proteins consists of small, evolutionarily conserved acute-phase proteins with important roles in inflammation, immunity, and lipid metabolism (1). SAA proteins respond rapidly to pro-inflammatory signals, such as cytokines like IL-1, IL-6, and TNFα. During inflammatory episodes, their plasma levels can increase over 1000-fold (2). In humans, there are four primary isoforms (SAA1-4): SAA1 and SAA2 are acute-phase proteins and most responsive to inflammation. These two SAAs are primarily expressed in the liver (3). SAA3, while functional in some animal species, is considered a pseudogene in humans. SAA4 is constitutively expressed at low levels in the liver, but maintains basal functions without major upregulation in response to inflammation (4). The four SAA isoforms participate in diverse processes, from High-Density Lipoprotein (HDL)-mediated lipid transport to innate immune signaling (5). The differential expression patterns and functional diversity among the isoforms highlight their adaptive roles in various physiological and pathological states, such as infections, chronic inflammatory diseases, and amyloidosis (6).

Notably, SAA proteins are emerging as significant biomarkers in precision medicine, particularly for predicting disease outcomes and personalizing treatment. In cancer, hepatocyte-derived SAA proteins are linked to immune evasion by reducing T-cell infiltration into tumors, influencing tumor progression and patient prognosis (7). Genetic predispositions also play an important role, as specific SAA gene variants increase susceptibility to chronic inflammatory diseases. In addition, high SAA protein levels are associated with relapse risk and treatment responses, aiding personalized care decisions in conditions like inflammatory bowel disease (8, 9). SAA protein levels are similarly valuable in acute infections and hyper-inflammatory states, where they indicate disease severity and guide tailored interventions, with COVID-19 being one such example (10). It has therefore been suggested that SAA proteins could serve as vital markers in infectious diseases including sepsis (11).

Under inflammatory conditions, SAA proteins play a pivotal role in influencing immune cell behavior and amplifying inflammatory signaling pathways. For instance, SAA1 has been shown to exacerbate airway inflammation by promoting neutrophil and macrophage activation, contributing to the pathogenesis of airway diseases (12, 13). In asthma, elevated SAA1 levels were found to drive airway hyperresponsiveness and increase the expression of inflammatory cytokines such as IL-6 and TNFα, which further amplifies the inflammatory response (14). Similarly, in kidney diseases, SAA2 has been reported to play a role in promoting podocyte injury through the activation of the NF-κB signaling pathway, highlighting its contribution to chronic inflammation in renal tissues (15).

Using a murine endotoxin model, SAA3 has been implicated in LPS-induced ocular inflammation, where it activates retinal microglia and promotes a pro-inflammatory state via Toll-like receptor 4 (TLR4) signaling. These results highlight the diverse cellular pathways that SAA proteins engage across different tissues (16). SAA proteins also affect lipid metabolism, as seen in patients with chronic inflammatory conditions, where SAA-modified high-density lipoprotein (HDL) particles lose their anti-inflammatory properties and become pro-inflammatory, further contributing to disease severity (17). Together the multifaceted role of SAA proteins in inflammation positions them as central mediators of both acute and chronic inflammatory diseases.

In the context of sepsis, SAA proteins have become increasingly recognized for their critical role in modulating the immune response through their rapid upregulation. Studies have demonstrated that patients with sepsis exhibit significantly higher levels of SAA proteins, which correlate with disease severity and the likelihood of developing septic shock (11). Therefore, elevated SAA protein levels are strongly associated with the severity of sepsis and are used as diagnostic and prognostic biomarkers. In particular, SAA protein levels have been shown to predict multiple organ dysfunction and mortality, making them valuable for assessing patient prognosis in the intensive care unit (ICU) (18).

The combined use of SAA proteins with other biomarkers, such as C-reactive protein (CRP) and procalcitonin (PCT), enhances diagnostic accuracy for early detection of sepsis, particularly in cases involving severe trauma or infections like urinary tract infections (19). In neonatal sepsis, point-of-care measurements of SAA levels have proven to be effective in early diagnosis, providing a faster and more accurate approach than traditional methods (20).

Moreover, role of SAA proteins extends beyond diagnosis to prognostication; studies have indicated that dynamic monitoring of SAA protein levels can help predict outcomes more reliably than other markers like nitric oxide or CRP alone (11). By closely tracking SAA protein levels over time, clinicians can better assess the severity of systemic inflammation, enabling more targeted therapeutic interventions to reduce mortality in septic patients.

This comprehensive role of SAA proteins in sepsis, from diagnosis to prognosis, underscores its potential as a crucial tool in the management of systemic inflammatory responses, particularly in critical conditions like septic shock.

The present study was undertaken to investigate the role of SAA proteins in modulating inflammatory responses under sterile and bacterial conditions. To this end, we stimulated bone marrow-derived macrophages (BMDMs) with a diverse panel of PAMPs and performed *in vivo* experiments using LPS-induced endotoxemia and bacterial infections.

## Materials and method

### Ethical statement

All experiments on mice were performed according to Swedish Animal Welfare Act SFS 1988:534 and were approved by the Animal Ethics Committee of Malmö/Lund, Sweden (permit numbers Dnr 5.8.18-18-01753/2022). Mice were housed in Innovive IVC Rodent Caging System on a 12/12 h light/dark cycle. The ambient temperature ranged between 19 and 23 °C with humidity 55  ±  10%. All animals had free access to water and chow. Technicians and staff did not enter the room during the dark cycle unless strictly required to collect cages for health monitoring. All animals received care according to the USA Principles of Laboratory Animal Care of the National Society for Medical Research, Guide for the Care and Use of Laboratory Animals, National Academies Press (1996) (46).

### Animals

*SAA^+/+^* (C57BL/6NJ) and *SAA^-/-^* (C57BL/6-Del(7Saa3-Saa2)738Lvh/J) mice (8 to 12 weeks of age, weighing 18 to 22 g (The Jackson laboratory, USA) were maintained under specific-pathogen-free conditions and had free access to commercial chow and water. *SAA^-/-^* mice genotype has been confirmed using a specific SAA ELISA.

### Isolation of bone marrow-derived macrophages

*SAA^+/+^* and *SAA^-/-^* knockout mice were sacrificed. The hind legs were removed and stored in HBSS + 1% BSA on ice. The femurs were extracted, and the upper and lower joints were cut using sterile scissors. The bone marrow was then flushed out with HBSS including 1% BSA solution using a syringe. The resulting cell suspension was passed through a 100 μm cell strainer into a new Falcon tube and centrifuged at 1600 rpm for 10 minutes. The supernatant was discarded, and 5 ml of sterile water was added for 5 seconds to osmotically lyse erythrocytes. The solution was then brought up to 50 ml with HBSS including 1% BSA and centrifuged again at 1600 rpm for 10 minutes. After discarding the supernatant, the cell pellet was resuspended in HBSS including 1% BSA, and the cells were counted. The cells were then transferred into T75 flasks containing 15 ml of DMEM medium (10% FBS, 1% Antibiotic-Antimycotic, and 50 mg/ml M-CSF).

### Plasmid restriction digestion and purification

The expression plasmids with Myc-DDK tags for SAA1 (MR200611, Saa1 (NM_009117) Mouse Tagged ORF Clone), SAA2 (MR219150, Saa2 (NM_011314) Mouse Tagged ORF Clone), SAA3 (MR200605, Saa3 (NM_011315) Mouse Tagged ORF Clone), and SAA4 (MR200725, Saa4 (NM_011316) Mouse Tagged ORF Clone) were purchased from Origene Technologies. BsrGI-HF^®^ (#R3575S) from New England Biolabs.

### Generation of SAA DDK stable macrophage cell lines

100,000 RAW Blue cells per well were seeded in a 24-well plate with 1 ml of DMEM (10% FBS, 1% Antibiotic-Antimycotic) per well. After 24 hours of incubation, NATE Transfection Enhancer (Cat. no: lyec-nate; Invivogen™) were added to each well and incubated for 30 minutes. Transfection was performed according to the instructions of the Lipofectamine LTX with Plus Reagent Kit (Cat. no: 15338100; Invitrogen™) to transfect the digested DDK-tagged SAA plasmids. For the selection of transfected cells after 48 hours after transfection, the plasmids containing G418 resistance genes were cultured in DMEM (10% FBS, 1% Antibiotic-Antimycotic, 2 mg/mL G418 (Cat. no: ant-gn-1; Invivogen™)) for 21 days. The medium with the added puromycin was changed every 3 days.

### Stimulation of macrophages

100,000 cells of each genotype were seeded in triplicates in a 96-well plate. The cells were stimulated with the reagents indicated in **Supplementary Table 1** or RAW cells stimulated with SAA proteins with or without LPS. After overnight incubation, the supernatants were collected and analyzed for cytokine levels.

### Stimulation of *SAA^+/+^* and *SAA^-/-^* KO BMDMs

100,000 cells of each genotype were seeded in triplicates in a 96-well plate. The cells were stimulated with the reagents indicated in **Supplementary Table 1** or RAW cells stimulated with SAA proteins (Nordic Biosite) with or without LPS. After overnight incubation, the supernatants were collected and analyzed for cytokine levels. The activators used in this study were chosen to model diverse inflammatory conditions. LPS, a TLR4 ligand, mimics sterile endotoxemia, while FSL-1 and Pam3CSK4, TLR2 agonists, represent bacterial and fungal infections. Zymosan, a β-glucan, was included to simulate sterile inflammation via TLR2 and dectin-1 pathways. These activators reflect key inflammatory contexts, enabling the evaluation of SAA-mediated immune modulation under both sterile and infectious conditions.

### Nuclear factor kappa B activity

After stimulation, RAW cells were spun down at 350 x g for 5 min and 20 µl of the supernatant was used for the NF-κB activation assay. After adding 180 µl of Quanti-Blue (Invivogen), samples were incubated at 37°C for 30 min and the absorbance was measured at 600 nm. Untreated cells served as a negative control. Cytokine heatmap was generated using the R package ComplexHeatmap (v2.22.0). Briefly, the mean of absorbance values (600nm) from three replicates (n=3) from different stimulations were obtained. The blank control value (n=3) was then subtracted from the means and ratios were taken between the stimulants versus untreated control samples (n=3). The ratios were then log2-transformed and presented in the heatmap.

### Murine endotoxemia model

SAA^+/+^ and SAA^-/-^ mice were injected intraperitoneally with LPS (8 mg/kg) in 200 µl PBS. Survival rates were monitored for 7 days. To determine cytokine levels, mice were sacrificed after 18 hours. Blood was collected via heart puncture into EDTA tubes, and mouse lungs were fixed in Histofix (Histolab Products AB, Gothenburg, Sweden) for immunohistochemistry. Blood samples were centrifuged at 1000 x g for 10 minutes at 4°C and stored at -80°C for further analysis.

### *Pseudomonas aeruginosa* infection mouse model

*P. aeruginosa* (strain Xen 41, derived from the parental pleural isolate *P. aeruginosa* PAO1; PerkinElmer, Waltham, MA) was grown aerobically in Todd-Hewitt (TH) broth at 37°C to the logarithmic growth phase (optical density at 620 nm [OD_620_] of ~0.5), harvested, washed in phosphate-buffered saline (PBS), and diluted to 2 x 10^9^ cfu/ml in the same buffer. Each animal was infected with 50 μl of the bacterial solution by intranasal instillation, whereas uninfected control animals received PBS alone. In order to maintain a consistent inoculum throughout the experiments, bacteria at a fixed OD_620_ nm value were used to infect the animals, and the cfu were confirmed by plating in every experiment. The mice were euthanized on the following day and the blood, BALF, lung, kidney, spleen and liver samples were collected for further processing. To quantify bactericidal activity, serial dilutions of the incubation mixtures were plated on TH agar, followed by incubation at 37°C overnight and determination of the number of cfu.

### Enzyme-linked immunosorbent assay

The ELISA was performed separately for SAA1, SAA2, SAA3, and SAA4 proteins using specific ELISA kits from Nordic Biosite. The following kits were used: Mouse SAA/SAA1 ELISA Kit (#KBB-9Y3TR5-96), Mouse SAA2 (Serum Amyloid A-2 Protein) ELISA Kit (#EKX-7XRF2Z-96), Mouse SAA3 (Serum Amyloid A-3 Protein) ELISA Kit (#EKX-4YOM8J-96), and Mouse SAA4 (Serum Amyloid A-4 Protein) ELISA Kit (#EKX-IZ7HDW-96). All four ELISAs followed a similar protocol. The protein concentrations were measured by using human antigen specific ELISA Kits according to manufacturer’s protocol. The absorbance was measured at 450 nm using a Victor Microplate Reader.

### Cytokine analysis using Bioplex

Cytokine levels in plasma and cell supernatants were analyzed using multiplex immunoassay kits (36-Plex ProcartaPlex Panel or 23-plex Bioplex Pro™, Invitrogen). The assays were performed according to the manufacturer’s instructions using a Bio-Plex™ 200 system (Bio-Rad).

### Hematoxylin and eosin staining

The left lungs were harvested in histofix, rehydrated, and embedded in paraffin. Sections of 4 μm thickness were placed on polylysine-coated glass slides, deparaffinized in histolab-clear (Histolab Products AB), rehydrated in graded alcohols and stained with hematoxylin and eosin by routine procedures.

### Statistical analysis

Experiments were done at least in two independent trials. Data were analyzed with GraphPad Prism 10.0 (GraphPad Software, San Diego, CA). All results are presented as mean values ± SEM with the number of independent experiments and mice per group indicated in the figure legends. Comparison of data was performed by one-way or two-way ANOVA and Tukey’s multiple comparison test.

## Results

### SAA proteins modulate cytokine and chemokine responses to diverse inflammatory stimuli

To explore the role of SAA proteins in modulating inflammatory responses, bone marrow-derived macrophages (BMDMs) from *SAA^+/+^* and *SAA^-/-^* mice were stimulated with a diverse set of PAMPs, including LPS, FSL-1, Pam3CSK4, and zymosan, to mimic bacterial, viral, fungal, and sterile inflammatory conditions. Cytokine and chemokine levels in the supernatant were quantified using multiplex immunoassays to assess the differential impact of SAA proteins on inflammatory signaling. Details about the specific ligands, their associated receptors, and the inflammatory contexts are summarized in **Supplementary Table 1**, providing a comprehensive overview of the experimental setup.

**Figure 1:**
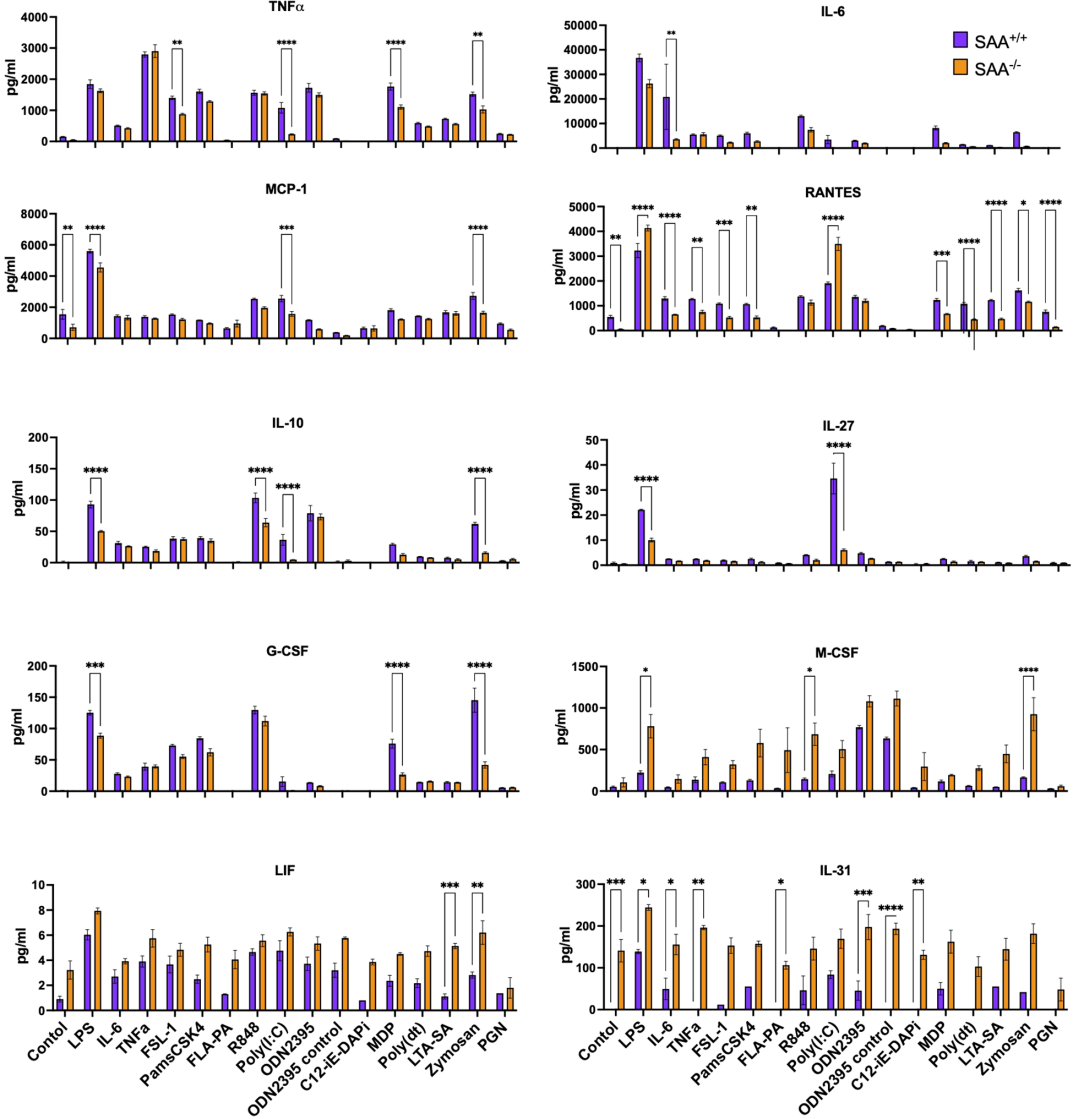
Measurement of cytokine and chemokine levels in response to stimulation with various immune ligands. *SAA^+/+^* and *SAA^-/-^* mice BMDMs were stimulated with a single dose of LPS, IL-6, TNFα, FSL-1, Pam3CSK4, FLAP2, R-848, Poly(I), ODN2395, C12-iE-DAP, MDP, Poly dT, LTA-SA, Zymosan, and Peptidoglycan overnight. Untreated BMDM cells served as controls. After overnight incubation of cells, the concentrations of TNFα, IL-6, MCP-1, RANTES, IL-10, IL-27, G-CSF, M-CSF, LIF, and IL-31 were determined. Significant differences between conditions are indicated by asterisks: *(p < 0.05), **(p < 0.01), ***(p < 0.001), and ****(p < 0.0001), one of the two representative experiment was presented (n=10 mice). A full list of the ligands, their corresponding receptors, and the type of infection they mimic (bacterial, viral, fungal, or sterile) is provided as **Supplementary Table 1**.

As shown in **Figure 1**, *SAA^+/+^* cells exhibited significantly elevated levels of pro-inflammatory cytokines, including TNFα, IL-6, MCP-1, and RANTES, compared to *SAA^-/-^* cells across most conditions. This effect was particularly pronounced with sterile inflammatory mimetics such as LPS, which elicited the highest cytokine responses. Other pro-inflammatory mediators, such as G-CSF and IL-31, also demonstrated significant genotype-dependent differences, though with greater variability across stimulators, suggesting that SAA proteins broadly enhance inflammatory pathways. In contrast, anti-inflammatory cytokines, including IL-10 and IL-27, displayed less consistent elevation between genotypes. This selective amplification of pro-inflammatory mediators highlights the role of SAA proteins in promoting robust immune activation in sterile inflammation while potentially maintaining balanced responses during bacterial challenges. Furthermore, immune mediators like LIF and M-CSF, which are associated with tissue repair and macrophage survival, were also elevated in *SAA^+/+^* cells under specific conditions, indicating potential additional roles for SAA proteins in cellular homeostasis during inflammation.

### NF-κB activation by SAA isoforms during sterile inflammation

To better understand the mechanisms underlying these observations, we investigated NF-κB activation in RAW 264.7 cells under various conditions. NF-κB is a pivotal transcription factor involved in the regulation of cytokine production and innate immune responses, particularly in response to microbial stimuli such as LPS (21). LPS was chosen because it is a well-characterized inducer of TLR4-mediated inflammatory responses, mimicking sterile endotoxemia and triggering the release of inflammatory mediators (22). RAW cells were either genetically modified to overexpress SAA genes (*SAA1*, *SAA2*, *SAA3*, and *SAA4*) or treated with purified SAA proteins, with and without LPS stimulation. These experiments aimed to assess whether SAA proteins directly or indirectly enhance NF-κB activity during sterile inflammation.

**Figure 2:**
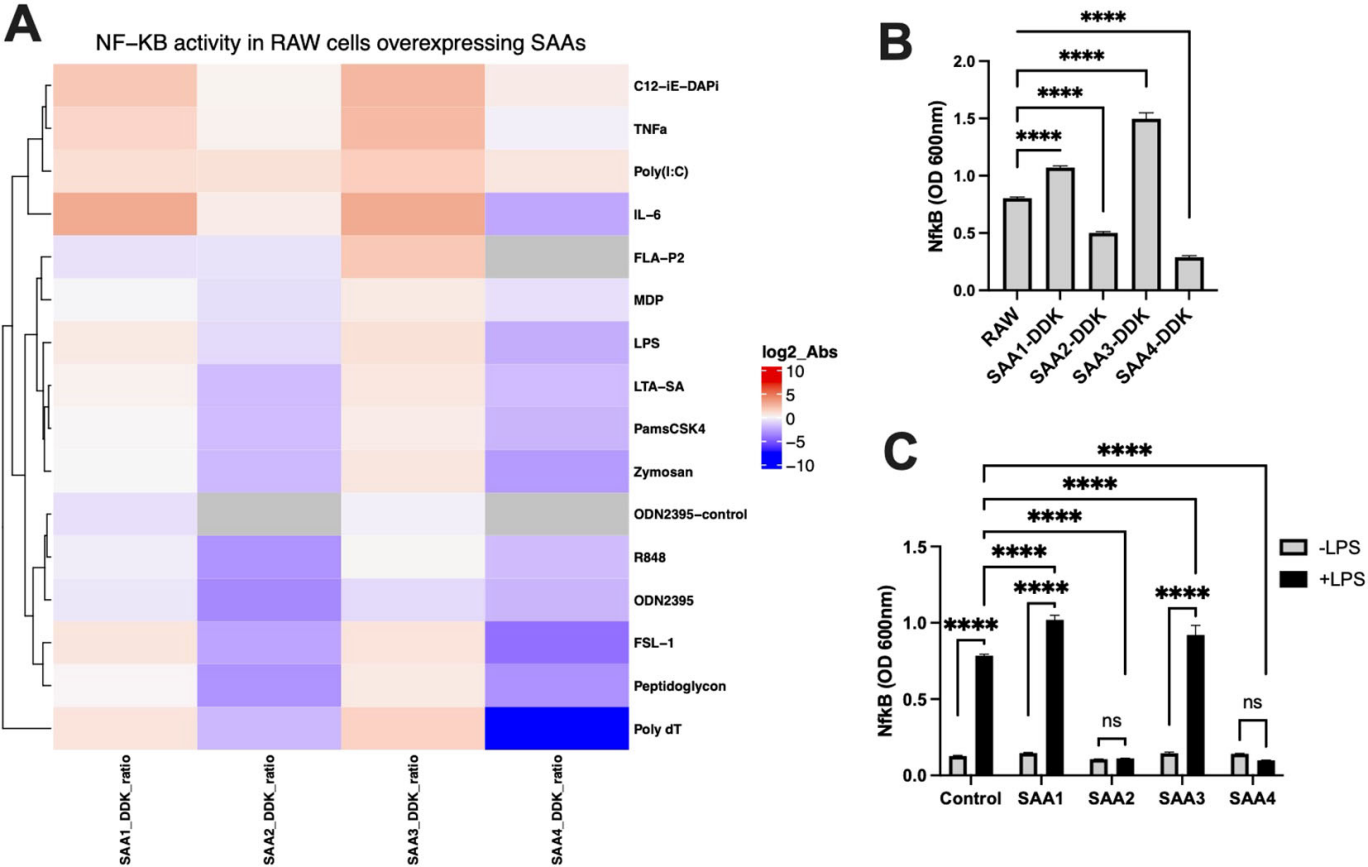
NF-κB activation in RAW cells overexpressing SAA proteins was assessed in response to stimulation with various immune ligands, or treatment with purified SAA proteins, compared to the response to LPS in control RAW cells. RAW-Blue™ cells (RAW 264.7) overexpressing SAA proteins were treated with a single dose of LPS, IL-6, TNFα, FSL-1, Pam3CSK4, FLAP2, R-848, Poly(I), ODN2395, C12-iE-DAP, MDP, Poly dT, LTA-SA, Zymosan, and Peptidoglycan overnight to activate NF-κB. Non-stimulated cells served as negative controls. **(A)** Heatmap showing NF-κB activity in RAW cells overexpressing SAA proteins (SAA1, SAA2, SAA3, SAA4) normalized against control RAW cells, with log2-transformed absorbance values (@600nm) displayed. **(B)** Representative bar graph of NF-κB activity in response to LPS stimulation in RAW cells overexpressing DDK-tagged SAA proteins (SAA1-DDK to SAA4-DDK) compared to unmodified RAW cells (n = 3). **(C)** NF-κB activity in RAW cells treated with purified SAA proteins (SAA1-100ng/ml, SAA2-10ng/ml, SAA3-10ng/ml, SAA4-100ng/ml) in the absence or presence of LPS (n = 3). Data represent mean ± SEM. Statistical significance is denoted as *(p < 0.05), **(p < 0.01), ***(p < 0.001), and ****(p < 0.0001). (ns = non-significant).

**Figure 2** illustrates the results of these experiments. **Figure 2A**, a heatmap shows NF-κB activity in RAW cells overexpressing different SAA isoforms. In the absence of agonists, NF-κB activity remained low across all groups. Upon different agonists stimulation, however, cells overexpressing SAA1 and SAA3 proteins exhibited significantly elevated NF-κB activity compared to SAA2 and SAA4 overexpressed cells. **Figure 2B**, a bar graph depicts NF-κB activity in RAW cells over-expressing DDK-tagged SAA isoforms. This finding aligns with previous reports that LPS activates TLR4-mediated pathways, with downstream activation of NF-κB as a key regulator of pro-inflammatory cytokine production (21, 23). Consistent with *Panel A*, all DDK-tagged SAA groups showed significant changes in NF-κB activity upon LPS stimulation compared to unmodified RAW cells. These results further validate the role of SAA proteins in modulating inflammatory signaling during sterile inflammation.

**Figure 2C** examines the effects of treating RAW cells with purified SAA proteins. Without LPS, purified SAA proteins did not significantly activate NF-κB, regardless of the isoform or concentration. However, under LPS stimulation, RAW cells treated with purified SAA1 and SAA3 proteins exhibited altered NF-κB activity compared to untreated RAW cells, whereas cells treated with SAA2 and SAA4 showed no significant changes in NF-κB activity due to stimulation. These findings underscore the dependency of SAA-driven NF-κB activation on microbial stimulation, reinforcing their role in amplifying sterile inflammatory responses (24, 25). Together our result show that SAA1 and SAA3 proteins amplify NF-κB activation and pro-inflammatory cytokine responses, whereas SAA2 and SAA4 proteins inhibit pro-inflammatory cytokine responses particularly under LPS-stimulated conditions.

### Survival and cytokine profiles following LPS challenge

To investigate the systemic implications of SAA proteins during sterile inflammation, we assessed survival and cytokine profiles in *SAA^+/+^* and *SAA^-/-^* mice following an intraperitoneal LPS injection (8 mg/kg). These experiments were designed to evaluate whether SAA proteins modulate survival and systemic inflammation under these conditions.

**Figure 3:**
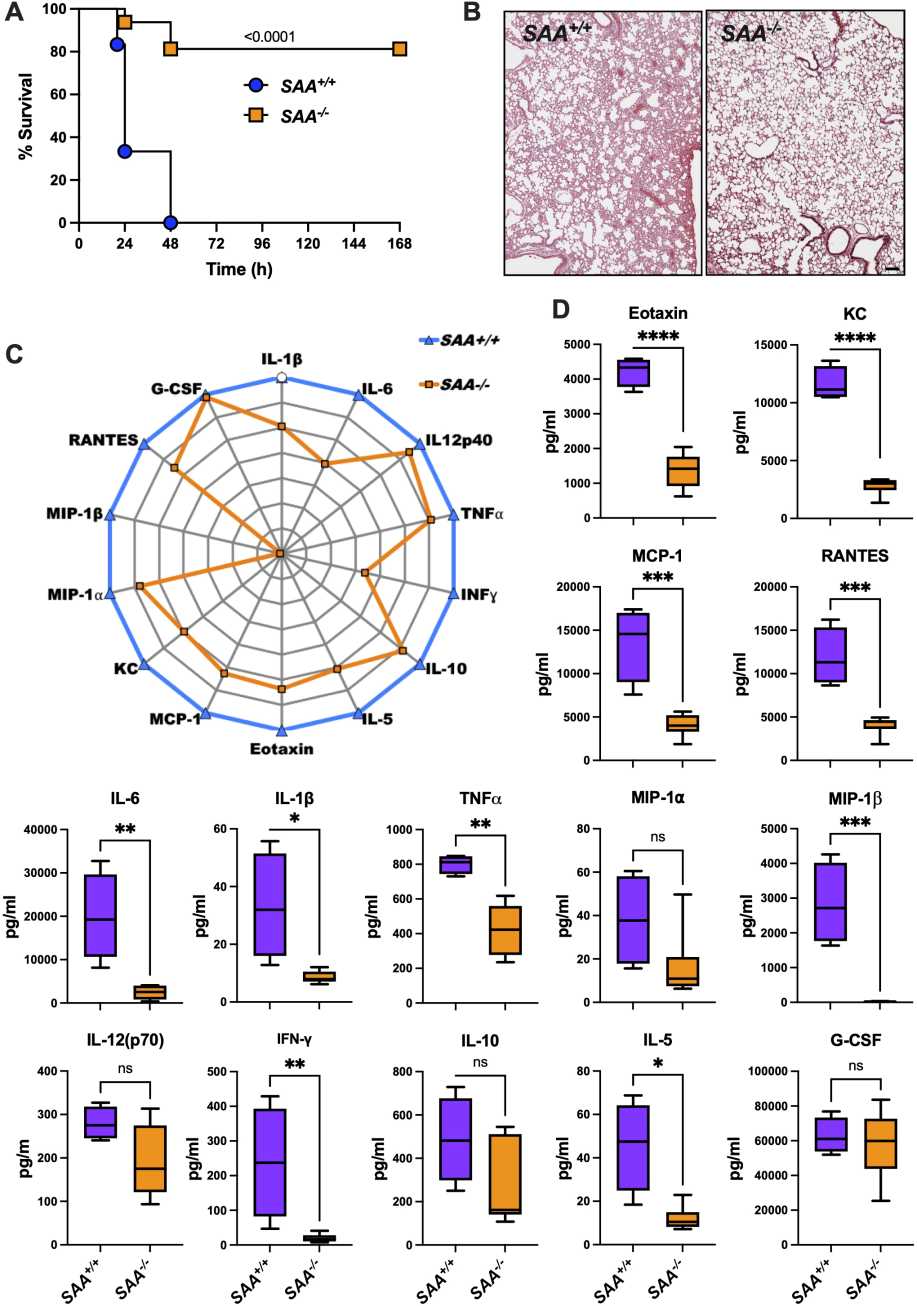
Survival, lung histology, and cytokine profiles in *SAA^+/+^* and *SAA^-/-^* mice following LPS challenge. **(A)** Kaplan-Meier survival curves of *SAA^+/+^* (n=6; blue) and *SAA^-/-^* (n=16; orange) mice i.p. injected with LPS (8 mg/kg). Mice were monitored over a 7-day period post-injection to assess survival rates. **(B)** Representative histological images of lung tissue from *SAA^+/+^* and *SAA^-/-^* mice 16 hours after LPS injection. Hematoxylin and eosin (H&E) staining shows tissue architecture, with each panel displaying one representative section from each genotype. **(C)** *SAA^+/+^* and *SAA^-/-^* mice were injected for 16 hours with LPS before blood was drawn. Normalized radar plot depicting the plasma levels of cytokines and chemokines, including IL-1β, IL-6, IL-12p40, TNFα, IFN-γ, IL-10, IL-5, Eotaxin, G-CSF, MCP-1, KC, MIP-1α, MIP-1β, RANTES, and G-CSF in *SAA^+/+^* (blue line) and *SAA^-/-^* (orange line) mice. Cytokine levels were measured 16 hours after LPS injection (*SAA^+/+^* (n=6) and *SAA^-/-^* (n=4)). **(D)** Box plots showing quantitative comparisons of cytokine concentrations (pg/ml) between *SAA^+/+^* (purple) and *SAA^-/-^* (orange) mice for Eotaxin, KC, MCP-1, RANTES, IL-6, IL-1β, TNFα, MIP-1α, MIP-1β, IL-12(p70). INF-γ, IL-10, IL-5 G-CSF. Data represent individual measurements with median and interquartile ranges indicated. Data are presented as mean ± SEM. Statistical significance is indicated by asterisks: *(p < 0.05), **(p < 0.01), ***(p < 0.001), and ****(p < 0.0001).

As shown in **Figure 3A**, *SAA^-/-^* mice exhibited significantly improved survival rates compared to *SAA^+/+^* mice over the 7-day observation period. This survival benefit highlights a potential inflammatory role of SAA proteins in mitigating lethal systemic inflammation.

To explore the underlying mechanisms, we quantified plasma levels of key cytokines and chemokines 16 hours post-LPS injection. **Figures 3C, D** reveal significantly elevated levels of pro-inflammatory mediators, including TNFα, IL-6, and IL-1β, in *SAA^+/+^* mice compared to *SAA^-/-^* mice. These cytokines are critical drivers of systemic inflammation, and their elevation suggests that SAA proteins amplify inflammatory signaling in response to LPS (11, 19). Interestingly, anti-inflammatory cytokines such as IL-10 showed no significant differences between genotypes, indicating a selective enhancement of pro-inflammatory pathways by SAA proteins.

Chemokine analysis demonstrated higher levels of MCP-1, KC, and RANTES in *SAA^+/+^* mice, supporting enhanced monocyte and neutrophil recruitment. Notably, eotaxin levels were also significantly elevated in *SAA^+/+^* mice, suggesting a broader regulatory role for SAA proteins in immune cell recruitment (14).

Histological analysis of lung tissue collected 16 hours after LPS injection (**Figure 3B**) showed less severe inflammatory damage in *SAA^-/-^* mice compared to *SAA^+/+^* mice. These observations suggest that SAA proteins not only amplify inflammatory signaling but may also play a role in protecting against end-organ damage during systemic inflammation. To conclude, the results demonstrate that SAA proteins enhance systemic inflammatory signaling while leading to a survival disadvantage in LPS-induced endotoxemia. This dual role highlights their importance in modulating inflammatory responses during sterile inflammation.

### Role of SAA proteins in bacterial clearance and cytokine modulation

**Figure 4:**
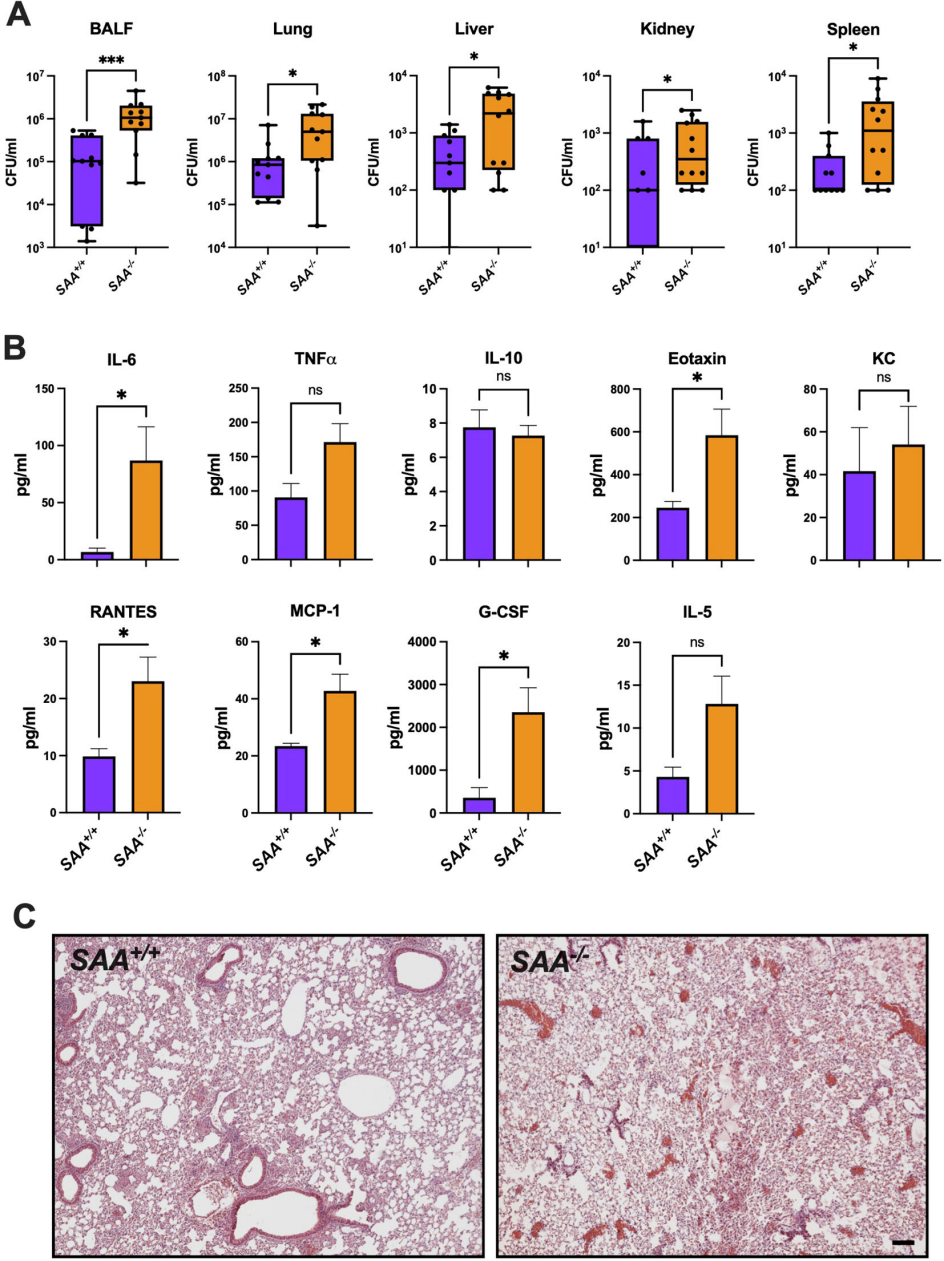
Bacterial burden, lung histology, and cytokine concentrations in *SAA^+/+^* and *SAA^-/-^* mice. *SAA^+/+^* and *SAA^-/-^* mice were infected i.n. with *P. aeruginosa* for (16 h). **(A)** Bacterial cfu counts in BALF, lung, liver, kidney, and spleen from *P. aeruginosa* infected *SAA^+/+^* and *SAA^-/-^* mice (n = 11) are shown. **(B)** The levels of inflammatory (IL-6, TNFα, RANTES, Eotaxin, KC, MCP-1, G-CSF, IL-5, IL-10) in *SAA^+/+^* (n = 4) and *SAA^-/-^* (n = 5) mice infected with *P. aeruginosa* were determined. **(C)** Representative lung histology sections (n = 3) from *SAA^+/+^* and *SAA^-/-^* mice treated with LPS are depicted. Data are presented as mean ± SEM. Statistical significance is indicated by *(p < 0.05), **(p < 0.01), ***(p < 0.001), and ****(p < 0.0001). (ns = non-significant).

To investigate the role of SAA proteins during bacterial infections, we evaluated bacterial clearance, cytokine responses, and organ-specific inflammatory changes in *SAA^+/+^* and *SAA^-/-^* mice following intranasal infection with *P. aeruginosa*. These experiments aimed to determine whether SAA proteins modulate immune responses to bacterial pathogens distinctively compared to sterile inflammation. As shown in **Figure 4A**, bacterial burden in the lungs of *SAA^+/+^* mice was significantly lower than in *SAA^-/-^* mice 24 hours post-infection, indicating enhanced bacterial clearance in the presence of SAA proteins. Similarly, bacterial counts were reduced in extrapulmonary organs, including the liver, spleen, and kidney, suggesting that SAA proteins play a systemic role in controlling bacterial dissemination.

Cytokine analysis of plasma revealed significantly higher levels of IL-6, TNFα, and MCP-1 in *SAA^-/-^* mice compared to *SAA^+/+^* mice (**Figure 4B**). These cytokines are key mediators of the innate immune response, facilitating pathogen clearance through immune cell activation and recruitment (26, 27). Interestingly, anti-inflammatory cytokines such as IL-10 exhibited no significant differences between genotypes, highlighting a pro-inflammatory bias associated with SAA proteins during bacterial infections. Histological examination of lung tissue (**Figure 4C**) revealed reduced inflammatory damage in *SAA^+/+^* mice compared to *SAA^-/-^* mice, characterized by less alveolar infiltration of neutrophils and fewer areas of necrosis. This suggests that SAA proteins may mitigate excessive tissue damage while supporting effective pathogen elimination. To summarize, these results highlight that SAA proteins enhance bacterial clearance and regulate inflammatory cytokine responses, facilitating pathogen elimination while minimizing severe tissue damage.

### Differential expression of SAA isoforms in response to inflammation and infection

**Figure 5:**
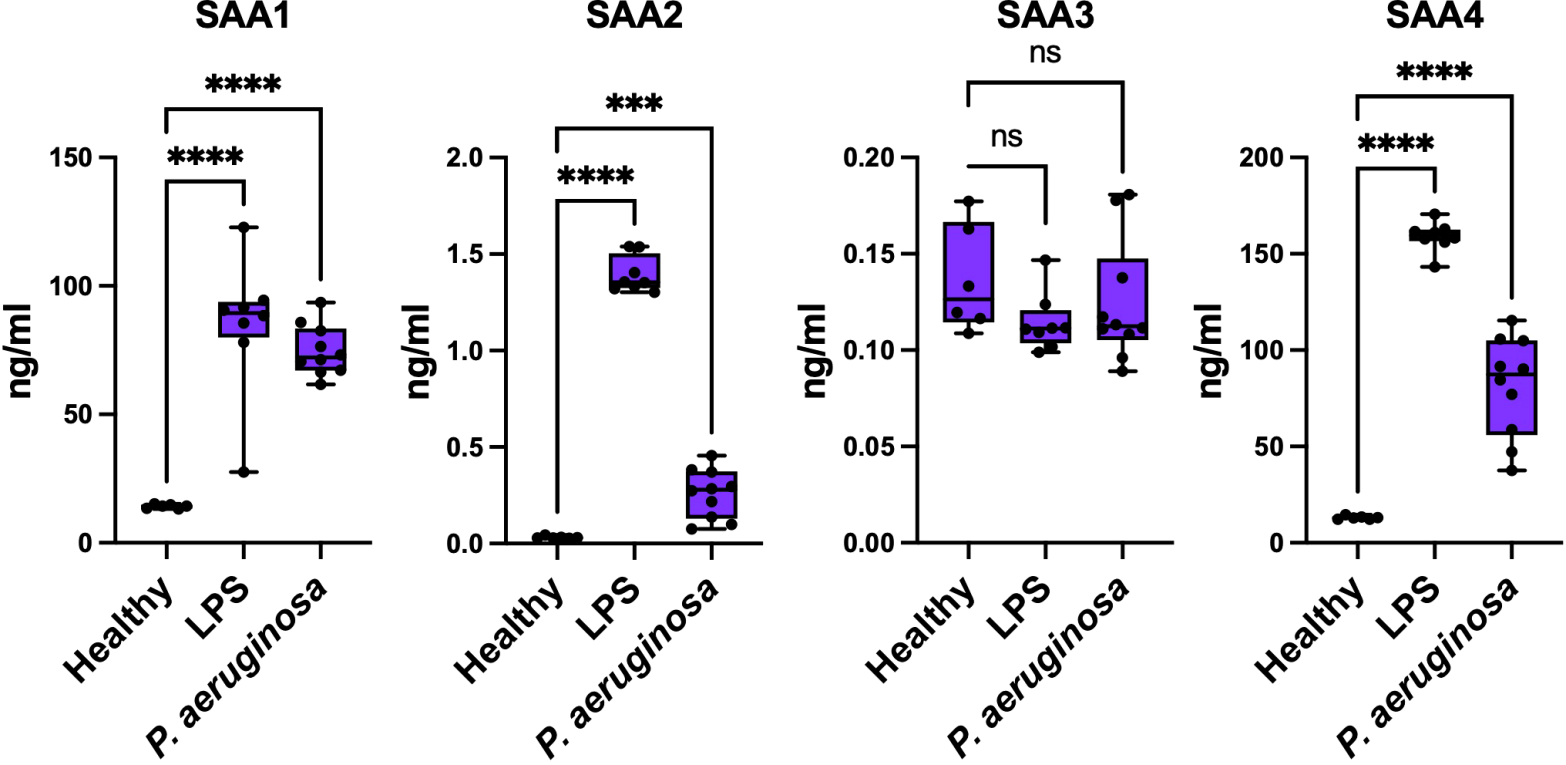
Concentrations of SAA proteins in response to LPS and *P. aeruginosa* infection. Concentrations of SAA1, SAA2, SAA3, and SAA4 were measured in samples from Healthy, LPS-treated, and *P. aeruginosa*-infected conditions (Healthy n = 6; LPS n=8; and *P. aeruginosa* n=10). Data represent mean ± SEM. Statistical significance is indicated by * (p < 0.05), ** (p < 0.01), *** (p < 0.001), and **** (p < 0.0001). (ns = non-significant).

To examine the expression patterns of SAA isoforms under different inflammatory conditions, we measured SAA1, SAA2, SAA3, and SAA4 levels in *SAA^+/+^* mice across three experimental groups: healthy controls, LPS-treated (sterile inflammation), and *P. aeruginosa*-infected (bacterial infection). These experiments aimed to determine whether distinct inflammatory stimuli elicit isoform-specific responses and how these differences contribute to systemic immune modulation. As shown in **Figure 5**, baseline SAA1-4 protein levels were undetectable in healthy control mice. Following LPS treatment, SAA1 and SAA2 levels were significantly elevated compared to baseline, reflecting their acute-phase response to sterile inflammation (28, 29). In contrast, SAA3 showed minimal induction, and SAA4 levels increased only modestly. In response to *P. aeruginosa* infection, SAA1 and SAA2 levels were also significantly upregulated but to a lesser extent than in the LPS-treated group. These findings suggest that SAA proteins respond more robustly to sterile inflammation than to bacterial infections, consistent with the notion that SAA1 and SAA2 are highly inducible under acute-phase conditions (30, 31).

These findings demonstrate that SAA1, SAA2 and SAA4 are key acute-phase responders, with their expression patterns varying significantly between sterile and bacterial inflammatory stimuli, reflecting their distinct roles in systemic immune regulation. Together, these findings reveal that SAA proteins play distinct roles in sterile and non-sterile inflammation, amplifying cytokine production and NF-κB activation in sterile conditions while promoting bacterial clearance and minimizing tissue damage during infections. This highlights their ability to tailor immune responses to specific inflammatory stimuli.

## Discussion

### Key findings

In this study, we investigated how SAA proteins modulate inflammatory responses during sterile endotoxemia and bacterial infections. Comprehensive *in vitro* and *in vivo* experiments were conducted to evaluate the effects of SAA proteins on cytokine and chemokine production, immune signaling pathways, and survival outcomes. Our findings demonstrate that SAA proteins exhibit context-dependent roles, amplifying inflammation in sterile conditions while promoting bacterial clearance during infection (32).

Understanding the distinct roles of SAA proteins in sterile and non-sterile inflammation is pivotal for bridging gaps in our knowledge of immune regulation and disease pathogenesis. Sterile inflammation, driven by damage-associated molecular patterns (DAMPs), underpins conditions such as sepsis, atherosclerosis, and autoimmune diseases, where unchecked immune responses lead to tissue damage and adverse clinical outcomes (32). In contrast, non-sterile inflammation, triggered by pathogen-associated molecular patterns (PAMPs) during microbial infections, requires a delicate balance between inflammatory responses and pathogen clearance to prevent systemic immune disruption (33). Positioned at the intersection of these inflammatory cascades, SAA proteins act as acute-phase reactants, orchestrating cytokine and chemokine networks to shape immune dynamics (34, 35).

### Mechanisms of action

To investigate how SAA proteins regulate inflammatory signaling, immune cell recruitment, and survival across these contrasting contexts, we conducted comprehensive *in vitro* and *in vivo* experiments. Our findings reveal that SAA proteins amplify cytokine and chemokine responses, a function particularly critical in sterile inflammation driven by DAMPs such as LPS (1). Cytokine and chemokine profiling as outlined in **Figure 1** demonstrates significantly higher levels of pro-inflammatory mediators, such as IL-6 and MCP-1, in *SAA^+/+^* macrophages compared to *SAA^-/-^* cells. These results reinforce the established role of SAA proteins in enhancing immune signaling networks (36) and align with previous studies linking proteins activity to immune cell recruitment during inflammatory responses (37).

Mechanistically, the activation of NF-κB by SAA proteins, as shown in **Figure 2**, offers valuable insights into how these acute-phase reactants amplify inflammatory signaling. NF-κB serves as a central regulator of pro-inflammatory cytokine production and plays a critical role in mediating sterile inflammation. The results demonstrate that RAW cells overexpressing SAA1 and SAA3 protein isoforms exhibited significantly enhanced NF-κB activity following LPS stimulation. Our finding aligns with previous reports suggesting that SAA proteins may not directly engage TLR4 but instead enhance its activation indirectly through interactions with LPS. Studies by others demonstrated that the binding of SAA proteins bind to LPS, can stabilize the LPS-TLR4 complex that amplifies downstream signaling pathways (38). This indirect mechanism provides a plausible explanation for the observed NF-κB activation in our study, particularly in the context of sterile inflammation where LPS is a primary agonist. To conclude, isoform-specific differences in NF-κB activation observed in our study point to potential specialization among SAA proteins, a hypothesis that warrants further investigation (39).

### Functional implications

Functionally, the survival analysis in LPS-induced endotoxemia, presented in **Figure 3**, provides compelling evidence for the protective role of SAA proteins in sterile inflammation.

*SAA^-/-^* mice exhibited significantly improved survival compared to their *SAA^+/+^* counterparts, likely reflecting a balance between enhanced immune activation and the mitigation of excessive tissue damage. This dual role of SAA proteins in amplifying inflammation while promoting its resolution, has also been observed in clinical studies linking SAA protein activity to better outcomes in sepsis and other inflammatory disorders (1). Collectively, these findings underscore SAA proteins as key modulators of inflammation, with distinct mechanisms tailored to sterile and infectious contexts. By integrating cytokine profiling, mechanistic insights, and survival outcomes, our results provide a comprehensive framework for understanding the dual functionality of SAA proteins and their therapeutic potential.

Building on these findings, our study further highlights the versatility of SAA proteins in adapting to distinct inflammatory demands. During sterile endotoxemia, SAA proteins amplify cytokine production, while in infectious contexts, they facilitate bacterial clearance. This evolutionarily conserved ability to modulate immune responses underscores their critical role as acute-phase reactants in maintaining immune homeostasis (40).

### Clinical implications

In sterile inflammation, the engagement of TLR4 by SAA proteins triggers NF-κB activation, driving the production of pro-inflammatory cytokines such as IL-6 and MCP-1 (41). This mechanism underscores the pivotal role of SAA proteins in sterile inflammatory pathways and its significance in systemic conditions such as sepsis and autoimmune diseases. Conversely, in infectious inflammation, SAA proteins interact with bacterial PAMPs to enhance immune cell recruitment and pathogen clearance. This dual role is exemplified by the opsonizing properties of SAA proteins, which promote phagocytosis and pathogen neutralization (42). Our findings on bacterial burden reduction in *SAA^+/+^* mice further support the role of SAA proteins in strengthening innate immune defenses (43).

The context-specific versatility of SAA proteins highlights their importance in balancing inflammatory amplification and resolution, reflecting a broader principle of immune homeostasis. These findings also emphasize the therapeutic potential of SAA proteins or its analogs, particularly in disorders like sepsis and bacterial infections. However, this adaptability warrants caution in therapeutic strategies; targeting SAA-TLR4 interactions or NF-κB signaling must preserve the protective functions of SAA proteins while mitigating excessive inflammation.

Future research should focus on isoform-specific targeting of SAA proteins to optimize therapeutic efficacy while minimizing potential risks. For instance, SAA1 and SAA2, which are robustly induced during inflammation, likely differ functionally from SAA4, whose constitutive expression suggests a distinct role in maintaining immune homeostasis (39). A deeper understanding of these isoform-specific functions could facilitate the development of precision therapies for inflammatory and infectious diseases.

### Limitations

Despite the valuable insights provided by our study into the roles of SAA proteins in sterile and bacterial inflammation, several limitations should be acknowledged. While murine models are indispensable for dissecting immune mechanisms, their relevance to human inflammatory responses requires further validation. Moreover, the focus on NF-κB signaling in this study leaves other pathways, such as JAK/STAT and MAPK, relatively underexplored. Additionally, the analysis was limited to specific cytokines and chemokines, and broader profiling or time-course studies could capture more comprehensive inflammatory dynamics. Direct evaluation of SAA protein-targeted interventions remains an important area for future research.

### Future directions

Future studies should focus on isoform-specific functions of SAA proteins, particularly SAA1 and SAA3, to clarify their distinct roles in immune regulation (24, 36). Investigating alternative pathways, such as JAK/STAT and RAGE, and validating findings in human systems will be critical for translating preclinical insights into clinical applications (44, 45). From a therapeutic perspective, targeting SAA protein-mediated pathways offers promising opportunities for managing inflammation in both sterile and infectious conditions. For instance, modulating TLR4-SAA protein interactions or developing isoform-specific inhibitors could mitigate excessive inflammation during sterile endotoxemia while preserving the protective functions of SAA proteins in bacterial clearance. Additionally, SAA proteins hold potential as biomarkers for assessing inflammation severity and therapeutic response, particularly in diseases such as sepsis and COVID-19, where precise immune modulation is essential (10). By addressing these gaps, future research could position SAA proteins as key targets for diagnostic and therapeutic innovations, providing novel strategies for managing inflammatory diseases.

## Data Availability

The raw data supporting the conclusions of this article will be made available by the authors, without undue reservation.

## References

[1] Sack GH. Serum amyloid A - a review. Mol Med. (2018) 24. doi: 10.1186/s10020-018-0047-0 PMC6117975 30165816

[2] Hosman IS Kos I Lamot L. Serum amyloid A in inflammatory rheumatic diseases: A compendious review of a renowned biomarker. Front Immunol. (2021) 11. doi: 10.3389/fimmu.2020.631299 PMC7933664 33679725

[3] Webb NR. High-density lipoproteins and serum amyloid A (SAA). Curr Atheroscler Rep. (2021) 23(2). doi: 10.1007/s11883-020-00901-4 PMC7808882 33447953

[4] Wakai M Hayashi R Ueno Y Onishi K Takasago T Uchida T. Promoting mechanism of serum amyloid a family expression in mouse intestinal epithelial cells. PloS One. (2022) 17(3). doi: 10.1371/journal.pone.0264836 PMC8932556 35303008

[5] Gursky O. Structural basis for vital function and malfunction of serum amyloid A: an acute-phase protein that wears hydrophobicity on its sleeve. Curr Atheroscler Rep. (2020) 22(11). doi: 10.1007/s11883-020-00888-y PMC7511256 32968930

[6] Zámocky M Ferianc P. Discovering the deep evolutionary roots of serum amyloid A protein family. Int J Biol Macromol. (2023) 252. doi: 10.1016/j.ijbiomac.2023.126537 37634776

[7] Stone ML Lee J Lee JW Coho H Tariveranmoshabad M Wattenberg MM. Hepatocytes coordinate immune evasion in cancer via release of serum amyloid A proteins. Nat Immunol. (2024) 25(5):755–63. doi: 10.1038/s41590-024-01820-1 PMC11186515 38641718

[8] Li ML Kim YM Koh JH Park J Kwon HM Park JH. Serum amyloid A expression in liver promotes synovial macrophage activation and chronic arthritis via NFAT5. J Clin Invest. (2024) 134(5). doi: 10.1172/JCI167835 PMC10904059 38426494

[9] Mestrovic A Perkovic N Bozic D Kumric M Vilovic M Bozic J. Precision medicine in inflammatory bowel disease: A spotlight on emerging molecular biomarkers. Biomedicines. (2024) 12(7). doi: 10.3390/biomedicines12071520 PMC11274502 39062093

[10] Almusalami EM Lockett A Ferro A Posner J. Serum amyloid A-A potential therapeutic target for hyper-inflammatory syndrome associated with COVID-19. Front Med-Lausanne. (2023) 10. doi: 10.3389/fmed.2023.1135695 PMC10060655 37007776

[11] Yu MH Chen MH Han F Li Q Sun RH Tu YX. Prognostic value of the biomarkers serum amyloid A and nitric oxide in patients with sepsis. Int Immunopharmacol. (2018) 62:287–92. doi: 10.1016/j.intimp.2018.07.024 30048858

[12] Bich TCT Quoc QL Choi Y Yang EM Trinh HKT Shin YS. Serum amyloid A1: A biomarker for neutrophilic airway inflammation in adult asthmatic patients. Allergy Asthma Immun. (2022) 14(1):40–58. doi: 10.4168/aair.2022.14.1.40 PMC8724823 34983106

[13] Zhou ZR Fang SB Liu XQ Li CG Xie YC He BX. Serum amyloid A1 induced dysfunction of airway macrophages via CD36 pathway in allergic airway inflammation. Int Immunopharmacol. (2024) 142. doi: 10.1016/j.intimp.2024.113081 39244902

[14] Yao XL Kaler M Qu X Kalidhindi RSR Sviridov D Dasseux A. Asthmatic patients with high serum amyloid A have proinflammatory HDL: Implications for augmented systemic and airway inflammation. J Allergy Clin Immun. (2024) 153(5). doi: 10.1016/j.jaci.2023.11.917 PMC10999351 38092139

[15] Lang YT Wang QM Sheng QH Lu SW Yang ML Kong ZJ. FTO-mediated m6A modification of serum amyloid A2 mRNA promotes podocyte injury and inflammation by activating the NF-κB signaling pathway. FASEB J. (2024) 38(2). doi: 10.1096/fj.202301419RR 38193628

[16] Feng HZ Zhou WC Yang Y Zhang XR Mao RX Zhou YT. Serum amyloid A aggravates endotoxin-induced ocular inflammation through the regulation of retinal microglial activation. FASEB J. (2024) 38(1). doi: 10.1096/fj.202301150RRR 38153347

[17] Peeran SW Elhassan A Zameer M Basheer SN Mustafa M Thiruneervannan M. Role of serum amyloid A protein in various diseases with special reference to periodontal and periapical inflammation- A review. J Clin Diagn Res. (2020) 14(12):Ze01–Ze5. doi: 10.7860/JCDR/2020/46072.14296

[18] Li M Qin YJ Zhang XL Zhang CH Ci RJ Chen W. A biomarker panel of C-reactive protein, procalcitonin and serum amyloid A is a predictor of sepsis in severe trauma patients. Sci Rep-Uk. (2024) 14(1). doi: 10.1038/s41598-024-51414-y PMC10770317 38182736

[19] Cui N Zhang YY Sun T Lv XW Dong XM Chen N. Utilizing procalcitonin, C-reactive protein, and serum amyloid A in combination for diagnosing sepsis due to urinary tract infection. Int Urol Nephrol. (2024) 56(7):2141–6. doi: 10.1007/s11255-024-03959-0 38376659

[20] Sharma V Grover R Priyadarshi M Chaurasia S Bhat NK Basu S. Point-of-care serum amyloid A as a diagnostic marker for neonatal sepsis. Indian J Pediatr. (2024) 91(6):571–7. doi: 10.1007/s12098-023-04677-8 37368220

[21] Martin MU Wesche H. Summary and comparison of the signaling mechanisms of the Toll/interleukin-1 receptor family. Bba-Mol Cell Res. (2002) 1592(3):265–80. doi: 10.1016/S0167-4889(02)00320-8 12421671

[22] Kuzmich NN Sivak KV Chubarev VN Porozov YB Savateeva-Lyubimova TN Peri F. TLR4 signaling pathway modulators as potential therapeutics in inflammation and sepsis. Vaccines-Basel. (2017) 5(4). doi: 10.3390/vaccines5040034 PMC5748601 28976923

[23] Millar MW Fazal F Rahman A. Therapeutic targeting of NF-κB in acute lung injury: A double-edged sword. Cells-Basel. (2022) 11(20). doi: 10.3390/cells11203317 PMC9601210 36291185

[24] den Hartigh LJ May KS Zhang XS Chait A Blaser MJ. Serum amyloid A and metabolic disease: evidence for a critical role in chronic inflammatory conditions. Front Cardiovasc Med. (2023) 10. doi: 10.3389/fcvm.2023.1197432 PMC10311072 37396595

[25] Chen XS Liu YC Gao YL Shou ST Chai YF. The roles of macrophage polarization in the host immune response to sepsis. Int Immunopharmacol. (2021) 96. doi: 10.1016/j.intimp.2021.107791 34162154

[26] Zheng H Li HF Zhang JY Fan HL Jia LN Ma WQ. Serum amyloid A exhibits pH dependent antibacterial action and contributes to host defense against cutaneous infection. J Biol Chem. (2020) 295(9):2570–81. doi: 10.1074/jbc.RA119.010626 PMC7049966 31819008

[27] Gatica S Fuentes B Rivera-Asín E Ramírez-Céspedes P Sepúlveda-Alfaro J Catalán EA. Novel evidence on sepsis-inducing pathogens: from laboratory to bedside. Front Microbiol. (2023) 14. doi: 10.3389/fmicb.2023.1198200 PMC10327444 37426029

[28] Kim MH de Beer MC Wroblewski JM Charnigo RJ Ji AL Webb NR. Impact of individual acute phase serum amyloid A isoforms on HDL metabolism in mice. J Lipid Res. (2016) 57(6):969–79. doi: 10.1194/jlr.M062174 PMC4878182 27018443

[29] Tang TZ Zhao YX Agarwal D Tharzeen A Patrikeev I Zhang YY. Serum amyloid A and mitochondrial DNA in extracellular vesicles are novel markers for detecting traumatic brain injury in a mouse model. Iscience. (2024) 27(2). doi: 10.1016/j.isci.2024.108932 PMC10844832 38323004

[30] Leung EL Lai HL Li RZ Pan HD Jiang ZB Li Y. Roles of serum amyloid A 1 protein isoforms in rheumatoid arthritis. Engineering-Prc. (2022) 10:174–82. doi: 10.1016/j.eng.2020.08.018

[31] Vollmer AH Gebre MS Barnard DL. Serum amyloid A (SAA) is an early biomarker of influenza virus disease in BALB/c, C57BL/2, Swiss-Webster, and DBA.2 mice. Antivir Res. (2016) 133:196–207. doi: 10.1016/j.antiviral.2016.08.011 27523492 PMC5042138

[32] Lin YK Zhang F Lei WJ Gan XW Li MD Pan F. Amnion-derived serum amyloid A1 participates in sterile inflammation of fetal membranes at parturition. Inflammation Res. (2023) 72(4):797–812. doi: 10.1007/s00011-023-01713-3 36879064

[33] Chait A den Hartigh LJ Wang SR Goodspeed L Babenko I Altemeier WA. Presence of serum amyloid A3 in mouse plasma is dependent on the nature and extent of the inflammatory stimulus. Sci Rep-Uk. (2020) 10(1). doi: 10.1038/s41598-020-66898-7 PMC7316782 32587356

[34] Su Q Weindl G. Glucocorticoids and Toll-like receptor 2 cooperatively induce acute-phase serum amyloid A. Pharmacol Res. (2018) 128:145–52. doi: 10.1016/j.phrs.2017.09.012 28941781

[35] Kamiya S Shimizu K Okada A Inoshima Y. Induction of serum amyloid A3 in mouse mammary epithelial cells stimulated with lipopolysaccharide and lipoteichoic acid. Animals-Basel. (2021) 11(6). doi: 10.3390/ani11061548 PMC8230092 34070499

[36] Ye RD Sun L. Emerging functions of serum amyloid A in inflammation. J Leukocyte Biol. (2015) 98:923–9. doi: 10.1189/jlb.3VMR0315-080R PMC6608020 26130702

[37] Li W Zhu S Li JH D’Amore J D’Angelo J Yang H. Serum amyloid A stimulates PKR expression and HMGB1 release possibly through TLR4/RAGE receptors. Mol Med. (2015) 21:515–25. doi: 10.2119/molmed.2015.00109 PMC4607615 26052716

[38] Zhou H Chen M Zhang G Ye RD. Suppression of lipopolysaccharide-induced inflammatory response by fragments from serum amyloid A. J Immunol. (2017) 199(3):1105–12. doi: 10.4049/jimmunol.1700470 28674180

[39] De Buck M Gouwy M Wang JM Van Snick J Opdenakker G Struyf S. Structure and expression of different serum amyloid A (SAA) variants and their concentration-dependent functions during host insults. Curr Med Chem. (2016) 23(17):1725–55. doi: 10.2174/0929867323666160418114600 PMC5405626 27087246

[40] Salama SA Gouwy M Van Damme J Struyf S. The turning away of serum amyloid A biological activities and receptor usage. Immunology. (2021) 163(2):115–27. doi: 10.1111/imm.v163.2 PMC8114209 33315264

[41] Yang SM Qin YM Ding L Wang JB Zhao HQ. Serum amyloid A aggravates lipopolysaccharide-induced injury of BEAS-2B cells by activating toll-like receptor 2/activator protein-1 signaling. J Biomater Tiss Eng. (2021) 11(2):282–9. doi: 10.1166/jbt.2021.2557

[42] Shah C Hari-Dass R Raynes JG. Serum amyloid A is an innate immune opsonin for Gram-negative bacteria. Blood. (2006) 108(5):1751–7. doi: 10.1182/blood-2005-11-011932 16735604

[43] Ji AL Trumbauer AC Noffsinger VP Meredith LW Dong B Wang Q. Deficiency of acute-phase serum amyloid A exacerbates sepsis-induced mortality and lung injury in mice. Int J Mol Sci. (2023) 24(24). doi: 10.3390/ijms242417501 PMC10744229 38139330

[44] Liu MJ Bao SY Napolitano JR Burris DL Yu LB Tridandapani S. Zinc regulates the acute phase response and serum amyloid A production in response to sepsis through JAK-STAT3 signaling. PloS One. (2014) 9(4). doi: 10.1371/journal.pone.0094934 PMC3986341 24732911

[45] Dong HB Zhang Y Huang Y Deng H. Pathophysiology of RAGE in inflammatory diseases. Front Immunol. (2022) 13. doi: 10.3389/fimmu.2022.931473 PMC9373849 35967420

[46] Worlein JM Baker K Bloomsmith M Coleman K Koban TL. The Eighth Edition of the Guide f or the Care and Use of Laboratory Animals (2011); Implications for Behavioral Management. Am J Primatol. (2011) 73:98–98.

