# Supplementary material for: Balancing inflammation: the specific roles of serum amyloid A proteins in sterile and infectious diseases
Source: Front Immunol. 2025 Feb 10;16:1544085. doi: 10.3389/fimmu.2025.1544085 (PMC11885894; doi:10.3389/fimmu.2025.1544085)
Supplement: Supplementary file 1 [file Table1.docx]

# Updated Table S1: Inflammatory Stimuli, Receptors, and Concentrations

| Ligand | Receptor | Inflammatory Context | Concentration |
| --- | --- | --- | --- |
| LPS | TLR4 | Bacterial | 1 μg/ml |
| C12-iE-DAP | NOD1 | Bacterial | 1 μg/ml |
| FLAP2 | TLR5 | Bacterial | 100 ng/ml |
| FSL-1 | TLR2/TLR6 | Bacterial | 0.1 ng/ml |
| LTA-SA | TLR2 | Bacterial | 500 ng/ml |
| MDP | NOD2 | Bacterial | 1 μg/ml |
| ODN2395 | TLR9 | Bacterial | 25 μg/ml |
| Pam3CSK4 | TLR1/TLR2 | Bacterial | 1 ng/ml |
| Peptidoglycan | TLR4 | Bacterial | 1 μg/ml |
| Poly(dT) | Cytosolic DNA | Viral | 5 μg/ml |
| Poly(I:C) | TLR3 | Viral | 10 μg/ml |
| R-848 | TLR7/TLR8 | Viral | 200 ng/ml |
| Zymosan | TLR2 | Fungal | 10 μg/ml |
| IL-6 | IL-6Rα | Sterile | 20 ng/ml |
| TNFα | TNFR1/TNFR2 | Sterile | 20 ng/ml |
